# Novel Mutations in *CLPP*, *LARS2*, *CDH23*, and *COL4A5* Identified in Familial Cases of Prelingual Hearing Loss

**DOI:** 10.3390/genes11090978

**Published:** 2020-08-22

**Authors:** Saba Zafar, Mohsin Shahzad, Rafaqat Ishaq, Ayesha Yousaf, Rehan S. Shaikh, Javed Akram, Zubair M. Ahmed, Saima Riazuddin

**Affiliations:** 1 Institute of Molecular Biology & Biotechnology, Bahauddin Zakariya University, Multan 60800, Pakistan; saba.zafar28@yahoo.com (S.Z.); ayesha_yousaf2007@yahoo.com (A.Y.); rehansadiq80@bzu.edu.pk (R.S.S.); 2 Department of Molecular Biology, Shaheed Zulfiqar Ali Bhutto Medical University, Islamabad 44000, Pakistan; mohsinzoologist@gmail.com (M.S.); zmahmed@som.umaryland.edu (Z.M.A.); 3 Department of Otorhinolaryngology Head and Neck Surgery, University of Maryland School of Medicine, Baltimore, MD 21201, USA; rafaqatishaq@gmail.com; 4 University Institute of Biochemistry & Biotechnology, PMAS-Arid Agriculture University, Rawalpindi 46000, Pakistan; 5 University of Health Sciences, Lahore 54600, Pakistan; vc@uhs.edu.pk

**Keywords:** prelingual hearing loss, genetic heterogeneity, whole-exome sequencing, genetic testing, Pakistan

## Abstract

We report the underlying genetic causes of prelingual hearing loss (HL) segregating in eight large consanguineous families, ascertained from the Punjab province of Pakistan. Exome sequencing followed by segregation analysis revealed seven potentially pathogenic variants, including four novel alleles c.257G>A, c.6083A>C, c.89A>G, and c.1249A>G of *CLPP*, *CDH23*, *COL4A5*, and *LARS2*, respectively. We also identified three previously reported HL-causing variants (c.4528C>T, c.35delG, and c.1219T>C) of *MYO15A*, *GJB2*, and *TMPRSS3* segregating in four families. All identified variants were either absent or had very low frequencies in the control databases. Our in silico analyses and 3-dimensional (3D) molecular modeling support the deleterious impact of these variants on the encoded proteins. Variants identified in *MYO15A*, *GJB2*, *TMPRSS3*, and *CDH23* were classified as “pathogenic” or “likely pathogenic”, while the variants in *CLPP* and *LARS2* fall in the category of “uncertain significance” based on the American College of Medical Genetics and Genomics/Association for Molecular Pathology (ACMG/AMP) variant pathogenicity guidelines. This paper highlights the genetic diversity of hearing disorders in the Pakistani population and reports the identification of four novel mutations in four HL families.

## 1. Introduction

Hearing loss (HL) is an etiologically heterogeneous trait that can present itself at any age and degree of severity. This condition affects 1 in 500 newborns and >360 million people worldwide [1,2]. Unlike genetic disorders caused by single-gene pathogenic variants (e.g., cystic fibrosis), over 120 distinct autosomal genetic loci are already linked to just the nonsyndromic form of recessively inherited HL [3]. It is estimated that up to 1% of human genes are essential for hearing function [4], and at least 1000 genes are associated with inherited HL, based upon studies on HL-associated diseases, unique inner-ear transcripts [5,6,7,8,9], and model organisms [10,11,12,13,14,15]. Intriguingly, of the 72 known nonsyndromic HL genes, 34 were initially identified in Pakistani families [3], and eventually, the variants in these genes were identified in populations around the world [16,17,18,19,20,21]. The Pakistani population is ideal for genetic studies because of its rich anthropogeneological background, via successive waves of invasions due to its pivotal location at crossroads of South Asia, the Middle East, and Central Asia, as well as its high consanguinity. Parental consanguinity accounts for a 0.25–20% higher chance of recessive genetic disorders [22]. Specific clans and high consanguinity in Pakistan provide a unique genetic resource (62.7% of marriages are consanguineous, of which ~80% are between first cousins) [23].

In the present study, we performed exome sequencing on the DNA samples of eight large consanguineous Pakistani families segregating prelingual HL. Four novel and three previously reported variants in seven known HL genes were identified, including five missense, one nonsense, and one frameshifting truncation allele. The results of this study further support the utility of exome sequencing and genetic screening of HL families to catalog the novel disease-causing variants of known genes, which will certainly aid in improving the clinical genetic diagnostic rate, as well as in establishing the frequency of previously reported alleles in the Pakistani population.

## 2. Materials and Methods

### 2.1. Subjects and Clinical Evaluation

All procedures in this study were approved by the Institutional Review Board (IRB) Committees (HP-00061036) of the University of Maryland School of Medicine, Baltimore, MD, USA; the Institute of Molecular Biology & Biotechnology, Bahauddin Zakariya University, Multan, Pakistan; and the Shaheed Zulfiqar Ali Bhutto Medical University, Islamabad, Pakistan. The tenets of the Declaration of Helsinki for human subjects were followed and informed written consent from adults and assent from minors was obtained from all the participating individuals prior to inclusion in the study. Family histories were taken from multiple members to establish family structure, comorbidities, the onset of disease, and treatment. Clinical phenotyping was performed through a detailed review of medical history, physical examination, pure tone audiometry, a tandem gait test, a Romberg test, and an ophthalmic examination. Genomic DNA was extracted from blood samples of participating individuals via an inorganic method [24].

### 2.2. Exome Sequencing and Bioinformatic Analyses

Exome sequencing was performed on probands of all families. Exome-enriched genomic libraries were prepared using the Agilent SureSelect Human Expanded All Exon V5 kit and sequenced on an Illumina HiSeq4000 with an average of 100× coverage. Data alignment, variant calling, and filtration were performed as described previously [25,26]. The Primer3 web resource (http://bioinfo.ut.ee/primer3-0.4.0/) was used to design primers for Sanger sequencing of the selected variants.

Clustal Omega (https://www.ebi.ac.uk/Tools/msa/clustalo/) multiple sequence alignment was used to appraise the evolutionary conservation of the identified variants. Mutation Taster (http://www.mutationtaster.org/), Polyphen-2 (http://genetics.bwh.harvard.edu/pph2/), Mutation Assessor (http://mutationassessor.org/r3/), SIFT (https://sift.bii.a-star.edu.sg/), and Combined Annotation Dependent Depletion score (https://cadd.gs.washington.edu/score) were used to evaluate the impact of the identified variants on the encoded proteins. Finally, the Varsome (https://varsome.com) online tool was used for the classification of HL-associated variants according to the American College of Medical Genetics and Genomics (ACMG) guidelines.

### 2.3. Structural Modeling

To further evaluate the impact of variants on secondary structure, 3D protein models were generated through the Phyre2 server (http://www.sbg.bio.ic.ac.uk/phyre2/html/page.cgi?id=index) and analyzed through the HOPE protein prediction tool (https://www3.cmbi.umcn.nl/hope/). The University of California, San Francisco (UCSF) CHIMERA online tool (https://www.cgl.ucsf.edu/chimera/) was used to visualize the impact of amino acid change on protein folding and ionic interactions.

## 3. Results

**Figure 1:**
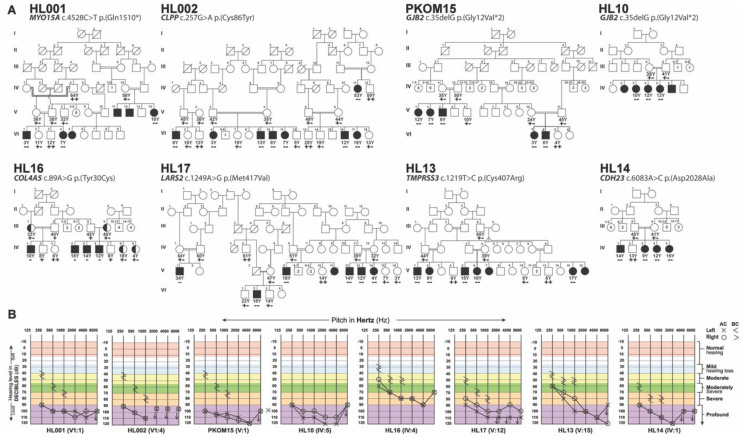
Hearing loss (HL) family pedigrees and causative variants. (**A**) Segregation of disease-causing alleles in eight Pakistani families. Filled and empty symbols represent affected and unaffected individuals, respectively, while half-filled symbols in family HL16 indicate carriers of identified X-linked variants. Double lines indicate consanguineous marriages. The genotypes (wild type, heterozygous, homozygous, or hemizygous) of the identified mutant alleles are also shown for each of the participating family members. All families had autosomal recessive mode of inheritance for HL, except for the family that had sex-linked (X-chromosome) inheritance. (**B**) Representative audiometric air (AC) and bone (BC) conduction thresholds from the affected individuals of eight Pakistani families revealed bilateral sensorineural hearing loss.

**Figure 2:**
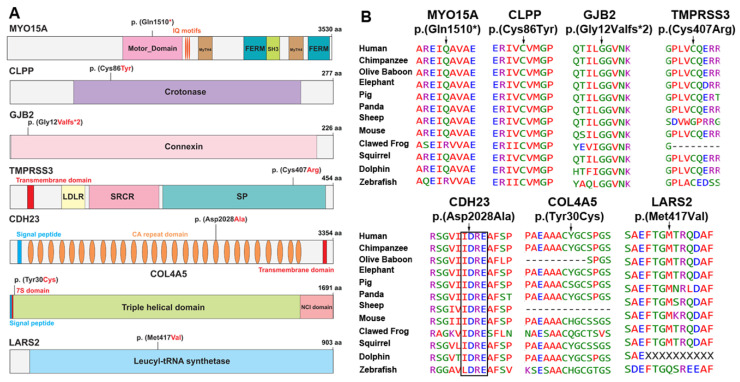
Protein structures and amino acid sequence alignments of orthologs. (**A**) Schematic representation of MYO15A, CLPP, GJB2, CDH23, COL4A5, and LARS2 proteins along with HL-associated variants identified in Pakistani families. (**B**) Clustal-W multiple amino acid sequence alignments of orthologous proteins showed evolutionarily conserved mutated residues across different species, except for the p.(Tyr30Cys) variant of COL4A5. However, none of the evaluated species had cysteine at position 30 in COL4A5 orthologs.

After IRB approval and informed consent, eight large consanguineous families (Figure 1A) were enrolled from the Punjab province of Pakistan (Figure 1A). According to family medical histories, all affected individuals had prelingual hearing loss (HL). Pure tone audiometric analysis revealed a bilateral mild to profound sensorineural hearing loss in all the tested individuals (Figure 1B). Consequently, to determine the genetic causes of HL segregating in these eight families, exome sequencing was performed for the proband of each family. Autosomal recessive inheritance, both homozygous and compound heterozygous, was assumed during the exome data filtering stages. We detected four novel variants, c.257G>A (p.(Cys86Tyr)), c.6083A>C (p.(Asp2028Ala)), c.89A>G (p.(Tyr30Cys)), and c.1249A>G (p.(Met417Val)), in *CLPP, CDH23, COL4A5,* and *LARS2*, and three previously reported variants, c.4528C>T (p.(Gln1510*)), c.35delG (p.(Gly12Valfs*2)), and c.1219T>C (p.(Cys407Arg)), in *MYO15A, GJB2,* and *TMPRSS3*, respectively (Figure 2A, Table 1). Except for the *COL4A5* allele, variants identified in this study were present in the evolutionarily conserved regions (Figure 2B) of the encoded proteins and were absent or had very low frequencies in the ExAC database (Table 1).

Next, to assess the predicted impact of identified HL-associated variants on the secondary structures of the encoded proteins, we performed 3D molecular modeling with Phyre2 and HOPE online programs. These models were generated using available structural information of the closely related proteins available in the NCBI protein database (https://www.ncbi.nlm.nih). The p.(Gln1510*) nonsense variant of myosin 15A and the p.(Gly12Val*2) frameshift variant of connexin 26 (encoded by *GJB2*), segregating with HL in three families, are likely to yield complete loss of function of both proteins, as the mRNAs harboring these alleles will likely be degraded through the nonsense-mediated decay (NMD) machinery [27]. In the unlikely event that *MYO15A* mRNA escapes NMD, the insertion of a nonsense codon at amino acid position 1510 is predicted to remove the carboxy tail, which will severely hamper the cargo function of the encoded protein [28,29].

**Figure 3:**
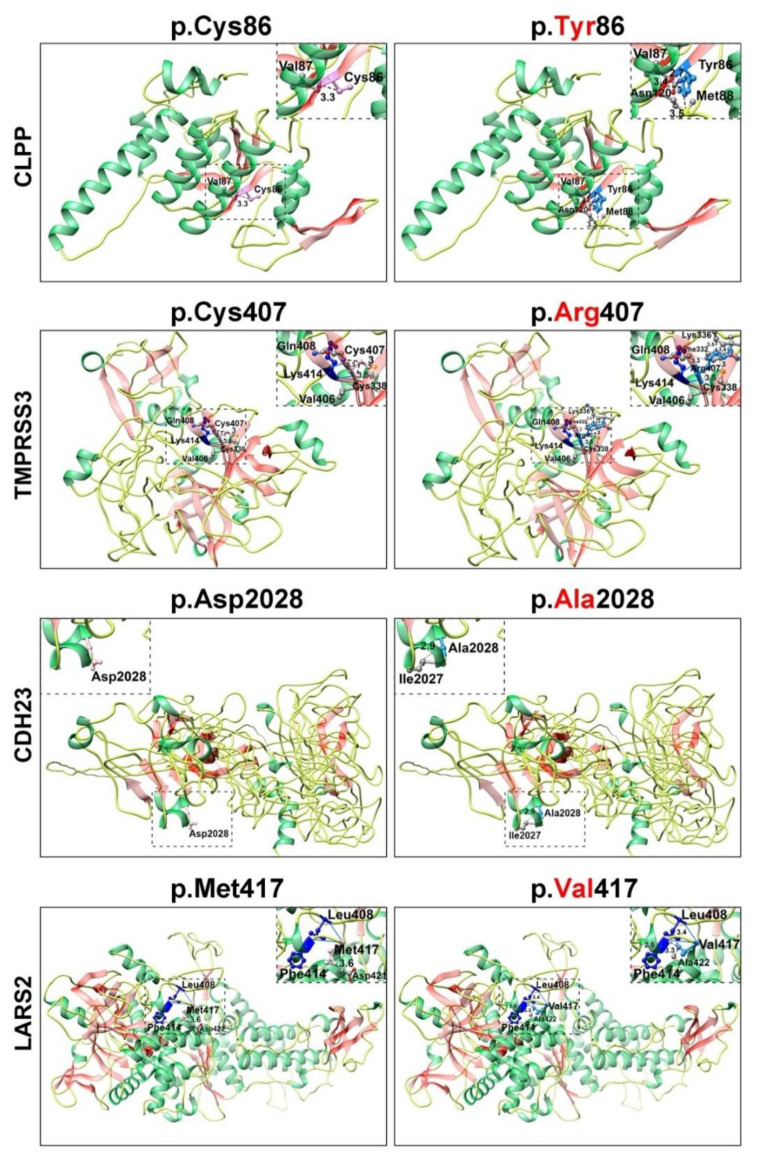
Protein 3D secondary structures generated by Phyre2 are shown in the respective colors: helix, green; strand, reddish pink; and coils, yellow. Pink and Dodger blue colors are used to show wild-type and mutant amino acids, respectively. Hydrogen bonding is shown by solid blue lines and concerned amino acids in dark blue color. Dotted lines represent the distance of the amino acids of interest with nearby residues in Angstroms respect. However, nearby residues are shown in color by element. The differences in size, charge, and hydrophobic properties of cysteine versus tyrosine at position 86 of CLPP might impact the interactions with other molecules on the surface of the protein. Similarly, the p.(Cys407Arg) missense substitution in TMPRSS3 is predicted to impact the core of the protein due to the larger size and different hydrophobic properties. The p.(Asp2028Ala) change mutates the calcium-binding motif (LDRE) of the cadherin repeat in CDH23, and causes a loss of interaction with the p.(Glu2030) residue. Finally, the p.(Met417Val) missense variant of LARS2 is predicted to induce aberrant ionic interactions with p.(Leu408).

The nonconservative p.(Cys86Tyr) variant of CLPP is predicted to change the torsion angle (Figure 3) since wild-type cystine is a sulfur-containing residue. This residue generally serves two essential biological roles: the site of redox reactions and participation in mechanical linkage for 3D folding of protein secondary structure. Replacement with tyrosine, which has a larger molecular size and different stereotypic properties, would likely impact protein folding and function (Figure 3). The p.Cys407 residue was located in the peptidase S1 enzymatic domain of TMPRSS3 and the p.(Cys407Arg) missense variant, found in family HL13, is predicted to cause loss of hydrophobic interactions in the core of the protein (Figure 3), leading to distortion of protein folding and thus abolishing the related function. The p.(Asp2028Ala) variant identified in family HL14 is predicted to alter the classical calcium-binding motif (LDRE; Figure 2B) within the cadherin repeat of CDH23, and is thus predicted to impair the calcium-binding ability (Figure 3). In contrast, the p.(Met417Val) variant, found in family HL17, was located in the transfer RNA (tRNA) synthetase domain of encoded leucyl-tRNA synthetase 2 (LARS2) protein. Replacement of methionine at position 417 with valine is predicted to alter the ionic interactions (hydrogen bonding) and folding of the secondary structure (Figure 3). Finally, the p.(Tyr30Cys) hemizygous variant of COL4A5, found in family HL16, could not be modeled due to lack of reasonable similarity to protein structures in the NCBI database. However, evaluation through the HOPE algorithm indicated that incorporation of a more-hydrophobic residue at position 30 could result in loss of hydrogen bonds and/or disturb the normal folding properties of COL4A5.

**Table 1:** Genes, identified variants, and their ACMG classification.

Family	Gene	cDNA Change	Protein Change	CADD	ExAC	Mutation Taster	Mutation Assessor	Polyphen 2	SIFT	ACMG Classification (Criteria Used)	Reference
HL001	*MYO15A*	c.4528C>T	p.(Gln1510*)	42	8 × 10^−6^	Disease causing	N/A	N/A	N/A	Pathogenic (PVS1, PM1, PM2, PP3, PP5)	[30]
HL002	*CLPP*	c.257G>A	p.(Cys86Tyr)	33	0	Disease causing	Low	Probably damaging	Damaging	Uncertain significance (PM2, PP3)	This study
HL10	*GJB2*	c.35delG	p.(Gly12Valfs*2)	N/A	0.006	Disease causing	Medium	Probably damaging	Damaging	Pathogenic (PVS1, PS3, PM1, PP3, BS2)	[31]
PKOM15	*GJB2*	c.35delG	p.(Gly12Valfs*2)	N/A	0.006	Disease causing	Medium	Probably damaging	Damaging	Pathogenic (PVS1, PS3, PM1, PP3, BS2)	[31]
HL13	*TMPRSS3*	c.1219T>C	p.(Cys407Arg)	27.5	0.00005	Disease causing	Medium	Possibly damaging	Tolerated	Pathogenic (PS1, PM1, PM2, PP2, PP3, PP5)	[32]
HL14	*CDH23*	c.6083A>C	p.(Asp2028Ala)	21.9	0.00001	Disease causing	High	Possibly damaging	Damaging	Likely pathogenic (PM1, PM2, PP3, PP5, BP1)	This study
HL16	*COL4A5*	c.89A>G	p.(Tyr30Cys)	22.8	0.0003	Benign	Neutral	Possibly damaging	Tolerated	Benign (PM1, PP2, BS1, BS2, BP4)	This study
HL17	*LARS2*	c.1249A>G	p.(Met417Val)	16.83	0.00002	Disease causing	Medium	Benign	Tolerated	Uncertain significance (PM2, PP2, BP4)	This study

N/A: Not applicable. CADD: Combined Annotation Dependent Depletion, https://cadd.gs.washington.edu/. ExAC: Exome Aggregation Consortium, http://exac.broadinstitute.org/. PVS1: pathogenic very strong (null variant (nonsense, frameshift, canonical ±1 or 2 splice sites, initiation codon, single or multiexon deletion) in a gene where loss of function is a known mechanism of disease)). PM1: pathogenic moderate 1 (located in a mutational hot spot and/or critical and well-established functional domain (e.g., active site of an enzyme) without benign variation). PM2: pathogenic moderate 2 (absent from controls (or at extremely low frequency if recessive) in Exome Sequencing Project, 1000 Genomes Project, or Exome Aggregation Consortium). PP3: pathogenic supporting 3 (multiple lines of computational evidence support a deleterious effect on the gene or gene product (conservation, evolutionary, splicing impact, etc.)). PP5: pathogenic supporting 5 (reputable source recently reports variant as pathogenic, but the evidence is not available to the laboratory to perform an independent evaluation). BP1: benign supporting 1 (missense variant in a gene for which primarily truncating variants are known to cause disease). BP4: benign supporting 4 (benign computational verdict because one benign prediction from GERP vs. no pathogenic predictions). BS1: benign supporting 1 (allele frequency is greater than expected for disorder). BS2: benign supporting 2 (observed in a healthy adult individual for a recessive (homozygous), dominant (heterozygous), or X-linked (hemizygous) disorder, with full penetrance expected at an early age).

## 4. Discussion

Advancements in molecular genetics screening and bioinformatics tools have been tremendously helpful in deciphering the causal variants for Mendelian disorders, including HL. Combinatorial approaches to identify individuals with actionable variants in highly penetrant genetic forms of common diseases like HL are essential if genomic medicine is to have its promised impact. With the advent of improved gene manipulation and delivery strategies to mitigate inherited HL [33,34,35,36], within the perceivable future, genetic testing will not only be useful for genetic diagnosis but also for personalized medicine. Here, we report the identification of seven HL-associated variants in eight multiplexed Pakistani families, including four novel alleles of *CLPP*, *LARS2*, *CDH23,* and *COL4A5* (Table 1). In addition, we also identified three previously reported variants of *MYO15A, GJB2*, and *TMPRSS3* in four large families (Figure 1A). All of these genes are highly expressed in the inner and outer hair cells of the cochlea [8,9], and their encoded protein products are required for the development, organization, maintenance, or ionic homeostasis of organ of Corti mechanosensory epithelia (e.g., [28,29]).

Affected individuals of families HL002 and HL17 were homozygous for the presumptive missense variants p.(Cys86Tyr) and p.(Met417Val) in CLPP and LARS2, respectively (Figure 1A). Biallelic variants in *CLPP* and *LARS2* are known to cause Perrault syndrome, a rare autosomal recessive disorder characterized by sensorineural HL in both sexes and primary ovarian failure in females [37,38]. In family HL002, four affected males and four affected females were found to have profound prelingual sensorineural HL. Affected female IV:11 (age 53 years) is currently at the menopausal stage; however, she and affected female IV:17 (age 19 years) were reported to have a history of normal menstrual cycles, although formal evaluation of hormonal profiles was not possible. Similarly, in family HL17, five affected males and two affected females were present. The only affected female that is still alive (V:14) has not reached puberty (age 4 years). Identification of a variant in *LARS2* that segregates in family HL17 is highly clinically relevant, considering that, without this genetic screening, the diagnosis of Perrault syndrome would not be considered in disease clinical management, prognosis, and counseling.

In family HL14, all the affected individuals were homozygous for a missense variant (p.(Asp2028Ala)) of CDH23 (Figure 1A). Biallelic variants in *CDH23* are a frequent cause of both nonsyndromic HL (DFNB12) as well as Usher syndrome type 1, an autosomal recessive disorder characterized by prelingual HL, vestibular areflexia, and progressive retinitis pigmentosa [39]. *CDH23* encodes a large protein with 27 extracellular calcium-binding cadherin motifs and a single transmembrane domain [39]. The p.(Asp2028Ala) variant identified in family HL14 is predicted to alter the classical calcium-binding motif (LDRE; Figure 2B) of the cadherin repeat. Mutations in the calcium-binding motifs often cause nonsyndromic HL with preserved retinal and balance functions [40,41]. Similarly, the affected individuals of family HL14 did not report any vision problems and appeared to have normal gait sophisticated function (evaluated through Romberg and Tandem gait tests). However, we cannot rule out the possibility that night vision problems, retinal degeneration, or balance areflexia might develop as these children age.Finally, in family HL16 with an X-linked HL inheritance pattern, we found a novel hemizygous missense variant p.(Tyr30Cys) of COL4A5 (Figure 1). As of March 2020, around 865 variants of *COL4A5* have been documented in the literature. They are known to cause Alport syndrome, a hereditary progressive kidney disease accompanied by ocular lesions and progressive or high tone sensorineural hearing loss [42]. However, currently, the affected individuals have no visual or renal problems. Parents of HL children with *COL4A5* variants should be made aware that alleles of this gene are associated with Alport syndrome. Subsequently, the parents should be offered genetic counseling to explain this potential outcome, and the children should undergo regular nephrological and ophthalmologic screening for kidney and ocular problems. In summary, for families living in remote areas of Pakistan with limited economic resources and sparse health facilities, genetic screening might further help in forming a complete diagnosis, enhancing family counseling, and advancing disease management.
